# LncRNA16 is a potential biomarker for diagnosis of early-stage lung cancer that promotes cell proliferation by regulating the cell cycle

**DOI:** 10.18632/oncotarget.13980

**Published:** 2016-12-16

**Authors:** Huange Zhu, Liyi Zhang, Shi Yan, Wenmei Li, Jiantao Cui, Min Zhu, Nan Xia, Yue Yang, Jiao Yuan, Xiaowei Chen, Jianjun Luo, Runsheng Chen, Rui Xing, Youyong Lu, Nan Wu

**Affiliations:** ^1^ Department of Thoracic Surgery II, Key Laboratory of Carcinogenesis and Translational Research (Ministry of Education/Beijing), Peking University Cancer Hospital and Institute, Beijing 100142, China; ^2^ Laboratory of Molecular Oncology, Key Laboratory of Carcinogenesis and Translational Research (Ministry of Education/Beijing), Peking University Cancer Hospital and Institute, Beijing 100142, China; ^3^ CAS Key Laboratory of RNA Biology, Institute of Biophysics, Chinese Academy of Sciences, Beijing 100101, China

**Keywords:** lncRNA16, biomarker, cyclin B1, lung cancer, cell cycle

## Abstract

Early diagnosis of lung cancer greatly reduces mortality; however, the lack of suitable plasma biomarkers presents a major obstacle. Recent studies showed that long noncoding RNAs (lncRNAs) played important roles in cancer initiation and development. Here, we identified differentially expressed lncRNAs in 20 lung cancer samples by using custom designed microarray and evaluated their expression in 118 lung cancer samples by real-time PCR (qRT-PCR). lncRNA16 (ENST00000539303) expression was significantly higher in lung cancer tissues (80/118) than in adjacent matched normal tissues. Importantly, this increase was similar to that in plasma (53/84) of lung cancer patients, including early stage. The role of lncRNA16 in lung cancer was studied *in vitro* and *in vivo* by using the lung cancer cell lines and xenograft mouse models. The results reveal that knockdown of lncRNA16 inhibited proliferation of PC9 cells *in vitro* and also inhibited tumor growth in xenograft mouse models. Overexpression of lncRNA16 promoted proliferation of A549 cells *in vitro* and also promoted tumor growth in xenograft mouse models. Specifically, we showed that lncRNA16 promoted G2/M transition by regulating cyclin B1 transcription. Together, our findings suggest that lncRNA16 is a promising biomarker suitable for early diagnosis of lung cancer, and a potential target for lung cancer treatment.

## INTRODUCTION

Lung cancer has been the leading cause of cancer-related mortality worldwide [1, 2]. The overall 5-year survival rate after curative tumor resection is relatively low in patients with lung cancer [3], mainly because it is usually detected at an advanced stage. Early diagnosis of lung cancer can improve a patient's prognosis and survival [4]; however, early diagnosis of this disease remains a challenge due to the lack of specific biomarkers in body fluids [5, 6].

Currently, methods for lung cancer diagnosis include the use of imaging and blood-fluid tests for tumor markers, both of which have limitations for early diagnosis. For example, screening with computerized tomography or low-dose computerized tomography often leads to over-diagnosis due to increased false positive results. Furthermore, the cumulative exposure to radiation associated with annual examinations represents a considerable health risk [7]. Thus, the application of such screening methods for routine lung cancer diagnosis is limited. Tests for blood-fluid tumor markers are widely employed in cancer diagnosis, and several cancer biomarkers, including carcinoembryonic antigen (CEA), cytokeratin 19 fragment (CYFRA21-1), and neuron-specific enolase (NSE) [8–10], are used to diagnose lung cancer. However, the sensitivity and accuracy of methods for detection of these biomarkers are relatively low. Rate of false-negative results while detecting these biomarkers is commonly higher than 50%. Thus, these markers lack sufficient sensitivity for reliable diagnosis of lung cancer at an early stage [11, 12]. Given these limitations, identification of new and more sensitive biomarkers for diagnosisis of early stage lung cancer is of great urgency.

Long noncoding RNA (lncRNAs) are more than 200 base pairs in length and lack protein-coding function [13, 14]. While playing important functions in the regulation of protein translation in healthy tissues, dysregulation of lncRNAs expression/function is thought to be an important cause of certain cancers. LncRNAs can act either as oncogenes or as tumor suppressors. For instance, steroid receptor RNA activator (SRA) is upregulated in breast cancer tissues, resulting in uncontrolled cell proliferation [15]. Hepatocellular carcinoma up-regulated long non-coding RNA (HULC) and HOXA distal transcript antisense RNA (HOTTIP) were previously reported to be highly expressed in hepatocellular carcinoma [16, 17]. Also, metastasis-associated lung adenocarcinoma transcript 1 (MALAT1) is highly expressed in lung cancer [18, 19]. In contrast, growth arrest-specific 5 (GAS5) is down regulated in breast cancer and hepatocellular carcinoma [20, 21], and maternally expressed 3 (MEG3) is downregulation in meningioma and colorectal cancer [22, 23], indicating that these two lncRNAs are involved in tumor suppression. Previous studies have shown that some of these lncRNAs could be used as biomarkers for specific types of cancers. For instance, plasma HULC was reported as a promising biomarker for hepatocellular carcinoma [24]. Moreover, with plasma levels of long intergenic non-protein-coding RNA 152 (LINC00152) significantly elevated in patients with gastric cancer, this lncRNA has the potential to serve as a blood-based biomarker during diagnosis of this disease [25]. Urinary prostate cancer associated 3 (PCA3) was also identified as a potential diagnostic indicator for prostate cancer, with upregulation of PCA3 in prostate cancer [26].

Here, lncRNA expression profiles in the lung cancer tissues and adjacent matched normal tissues were compared using an lncRNA microarray, and the differential expression of lncRNAs was investigated [27]. In summary, we identified several lncRNAs that were highly expressed in lung cancer tissues as compared to the normal tissues. In particular, lncRNA16 was highly expressed in the lung cancer tissues and plasma, and was detected at different stages of the disease, including the early stage. Furthermore, we studied the function and preliminary mechanism of action of lncRNA16 in a lung cancer cell line.

## RESULTS

### Expression of lncRNA16 is increased in the lung cancer tissues

A total of 138 primary lung cancer samples were used to identify and confirm the potential lung cancer biomarkers through custom designed microarray and subsequently confirmation. In the previous study, we used a custom microarray platform to constructed lncRNA expression profile in 20 samples each of 4 cancer types— lung cancer, liver cancer, colon cancer, and gastric cancer.

A series of lncRNAs, which were higher expressed in lung cancer compared with adjacent matched normal tissues, were identified. The expression of all the lncRNA in four cancer types (including lung cancer, liver cancer, colon cancer, and gastric cancer) detected by microarray was validated by Reverse transcriptase PCR (RT-PCR). The results revealed that differential expression of ENST00000539303 (we termed it as lncRNA16) was detected only in lung cancer, but not in other cancers (data not shown).

**Figure 1:**
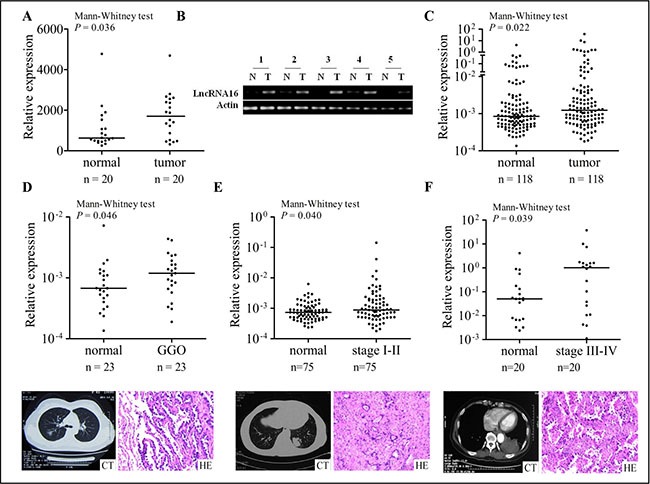
Increased tissue levels of lncRNA16 in lung cancer tissue samples (**A**) Relative expression of lncRNA16 in 20 lung cancer tissues and adjacent matched normal tissues was analyzed using an lncRNA microarray. (**B**) LncRNA16 expression in five lung cancer tissues and adjacent matched normal tissues was measured by RT-PCR. T, tumor tissues; N, adjacent matched normal tissues. (**C**) Relative lncRNA16 expression in 118 lung cancer tissues and adjacent matched normal tissues was determined by qRT-PCR. (**D**) Relative lncRNA16 expression in 23 GGO and adjacent matched normal tissues. The classic characters of radiology and pathology were ovserved by CT and H&E staining. (**E**) Relative lncRNA16 expression in 75 early stage lung cancer tissues and adjacent matched normal tissues. The classic characters of radiology and pathology were ovserved by CT and H&E staining. (**F**) Relative lncRNA16 expression in 20 advanced stage lung cancer tissues and adjacent matched normal tissues. The classic characters of radiology and pathology were showed by CT and H&E staining.

As shown in Figure 1A, the expression of lncRNA16 was significantly higher in the lung cancer tissues than in adjacent matched normal tissues and the data were confirmed by using RT-PCR (Figure 1B). Based on this observation, we verified the expression of lncRNA16 by real-time PCR (qRT-PCR) for an additional 118 lung cancer tissue and adjacent matched normal tissue samples. LncRNA16 expression was found to be significantly higher in the lung cancer tissues than in adjacent matched normal tissues (Figure 1C). According to the clinical stage of lung cancer, these samples were further divided into three groups: 23 samples in the ground glass opacity (GGO) group, representing one of the earliest stages of lung cancer; 75 in the early stage (including stage I and II) cancer group; and 20 in the advanced stage (including stage III and IV) cancer group. In all the groups, lncRNA16 expression was significantly higher in the lung cancer tissues than in adjacent matched normal tissues (Figure 1D–1F), indicating that lncRNA16 was already upregulated in the earliest stages of lung cancer.

### Expression of lncRNA16 is increased in plasma of patients with lung cancer

**Figure 2:**
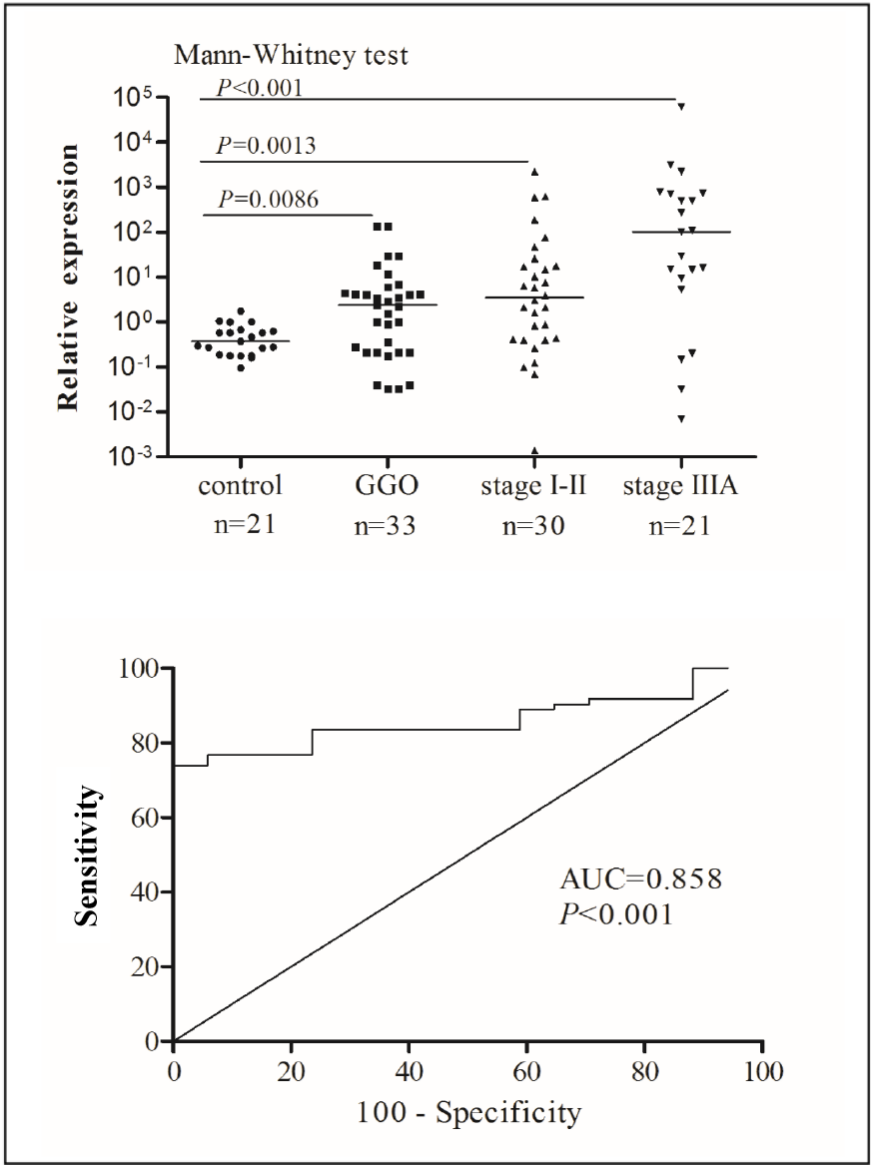
Increased plasma levels of lncRNA16 in patients with lung cancer (**A**) LncRNA16 expression levels in plasma of patients with lung cancer and non-cancer participants was analyzed by qRT-PCR. (**B**) To distinguish patients with lung cancer from non-cancer participants, diagnostic efficacy of plasma lncRNA16 was evaluated using ROC curve analysis.

To test whether lncRNA16 is a suitable plasma biomarker, we measured the lncRNA16 levels in the plasma of patients with lung cancer and compared it to those in the plasma of non-cancer participants. The analysis included 33 patients in the GGO group, 30 in the early-stage lung cancer group, and 21 in the advanced stage lung cancer group. As shown in Figure 2A, in each group, the lncRNA16 level was significantly higher in the plasma of patients with lung cancer than in plasma of non-cancer participants.

Next, the diagnostic performance of lncRNA16 was examined by performing a receiver operator characteristic (ROC) curve analysis of plasma lncRNA16. The results showed that lncRNA16 was effective for diagnosing lung cancer, as indicated by the area under the ROC curve (AUC-ROC) which was 0.858 (*P* < 0.001) (Figure 2B). With a cut-off value of 1.945 (relative lncRNA level calculated by 2^–ΔCT^), the sensitivity and specificity for distinguishing lung cancer samples from normal samples was 73.97% and 100.0%, respectively, indicating a drastic improvement over existing biomarkers. Further, to assess the diagnostic value of plasma lncRNA16, plasma lncRNA16 levels were compared with those of CEA, CA199, CA125, NSE, CYFRA21-1 and SCC, markers widely used for lung cancer assessment. As shown in Table 1, the rates of lncRNA16 positive detection was higher than that for these markers (*P* < 0.001). Together, our data convincingly showed that plasma levels of lncRNA16 accurately reflected the disease status of the patient, thus rendering this lncRNA an ideal candidate for diagnosis of lung cancer.

**Table 1:** The rate of lncRNA16 positive detection in plasma compared to that of other markers for diagnosis of lung cancer

	Total	Positive (%)	Negative (%)	*P*
CEA	76	18 (13.68)	58 (76.32)	< 0.001
CA199	73	5 (6.85)	68 (93.15)	
CA125	73	2 (2.74)	71 (97.26)	
NSE	73	31 (42.47)	42 (57.53)	
CYFRA21-1	73	13 (17.81)	60 (82.19)	
SCC	73	5 (6.85)	68 (93.15)	
lncRNA16	84	54 (64.29)	30 (35.71)	

Abbreviations: CEA, carcinoembryonic antigen; CA199, carbohydrate Atigen 19–9; CA125, cancer antigen 125; NSE, neuron-specific enolase; CYFRA21-1, cytokeratin 19 fragment; SCC, squamous cell carcinoma antigen.

Normal range: CEA: 0–5 ng/ml; CA199: 0–37 U/ml; CA125: 0–35 U/ml; NSE: 0–15.2 ng/ml; CYFRA21-1: 0–3.3 ng/ml; SCC: 0–1.5 ng/ml; LncRNA16: 0–0.0008196.

### lncRNA16 promotes cell proliferation *in vitro* and *in vivo*

**Figure 3:**
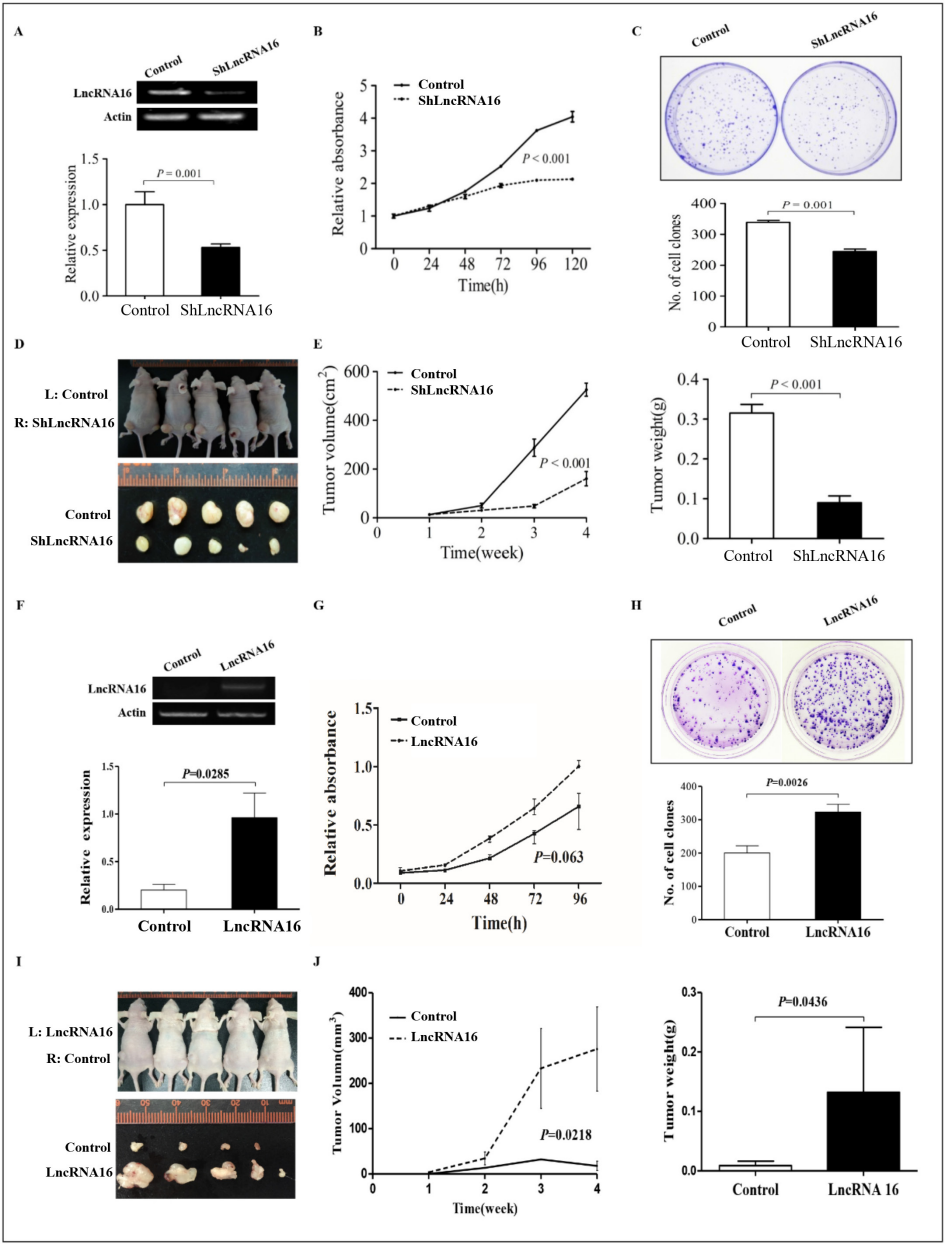
LncRNA16 promotes cell proliferation in vitro and in vivo (**A**) RT-PCR and qRT-PCR revealed that lncRNA16 level in PC9-shLncRNA16 cells was reduced compared to that in control (empty vector) cells. (**B**) MTT assay showed decreased cell proliferation in PC9-shLncRNA16 cells compared to control cells. (**C**) The ability for colony formation was significantly lower in PC9-shLncRNA16 cells than in control cells. (**D**) Images of tumors four weeks post injection of PC9-shLncRNA16 and control cells. L, left; R, right. (**E**) left, tumor volumes at various times points post injection of PC9-shLncRNA16 and control cells. E right, tumor weights noted four weeks post injection of PC9-shLncRNA16 and control cells. (**F**) RT-PCR and qRT-PCR revealed that lncRNA16 levels in A549-LncRNA16 cells were increased compared to control (empty vector). (**G**) MTT assay revealed increased cell proliferation in A549-LncRNA16 compared to that in control cells. (**H**) The ability for colony formation was significantly higher in A549-LncRNA16 cells than in control cells. (**I**) Images of tumors four weeks post injection of A549-LncRNA16 and control cells. L, left; R, right. (**J**) left, tumor volumes at various times points post injection of A549-LncRNA16 and control cells. J right, tumor weights noted four weeks post injection of A549-LncRNA16 and control cells.

To investigate the effects of lncRNA16 on cell proliferation, knockdown of lncRNA16 was done by shRNA. As shown in Figure 3A, the level of lncRNA16 in PC9-shLncRNA16 cells was significantly reduced compared to that in control (empty vector) cells. Furthermore, knockdown of lncRNA16 significantly inhibited cell growth (Figure 3B) and clone formation (Figure 3C), as revealed by MTT and colony-formation assay, respectively.

The function of lncRNA16 *in vivo* was investigated by injecting control and PC9-shLncRNA16 cells subcutaneously into nude mice. Four weeks post injection, we found that PC9-shLncRNA16 xenograft tumors were significantly smaller than control xenograft tumors (Figure 3D). A difference between in tumor volume was observed between the two groups two weeks post injection, and the difference was significant at week three and four (Figure 3E left). The mice were sacrificed four weeks post injection and the tumors were weighted. The results showed that tumors grown from PC9-shLncRNA16 cells were significantly smaller and lighter than those grown from control cells (Figure 3E right).

To validate the effects of lncRNA16 on cell proliferation, lncRNA16 was overexpressed in A549 cell line. As shown in Figure 3F, the level of lncRNA16 in A549-LncRNA16 cells was significantly increased compared with that in control (empty vector) cells. Furthermore, overexpression of lncRNA16 significantly promoted cell growth (Figure 3G) and clone formation (Figure 3H), as revealed by MTT and colony-formation assay, respectively.

The function of lncRNA16 was investigated *in vivo* by injecting control and A549-LncRNA16 cells subcutaneously into nude mice. Four weeks post injection, we found that A549-LncRNA16 xenograft tumors were significantly larger than control xenograft tumors (Figure 3I). A difference in tumor volume between the two groups was observed two weeks post injection, and a significant difference was observed at week three and four (Figure 3J left). At four weeks post injection, the mice were sacrificed and the tumors were weighted. The results showed that tumors grown from A549-LncRNA16 cells were significantly bigger than those grown from control cells (Figure 3J right).

**Figure 4:**
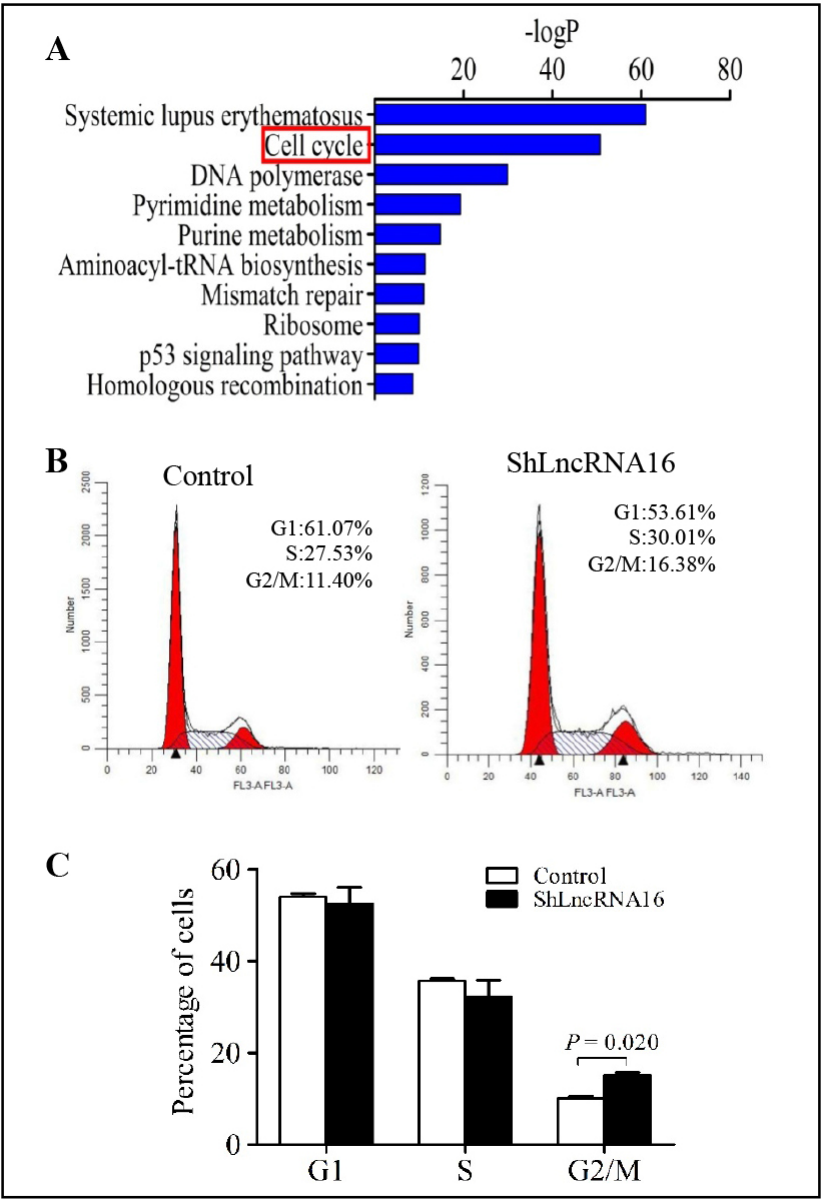
LncRNA16 knockdown in PC9 cells induces G2/M arrest (**A**) Pathways associated with lncRNA16 were analyzed using a bioinformatics approach. (**B**) and (**C**) Cell cycle analysis of PC9-shLncRNA16 and control cells was performed using flow cytometry. The results are presented as mean ± SD of a triplicate assay.

To investigate the mechanisms involved in lncRNA16-knockdown-meditated inhibition of cell growth, a bioinformatics approach was used to analyze the pathways associated with lncRNA16. The results revealed that one of the biological processes associated with lncRNA16 was related to cell cycle control (Figure 4A).

To test this association, we analyzed the impact of lncRNA16 on the progression of the cell cycle. Compared to control experiments, knockdown of lncRNA16 induced G2/M phase arrest (Figure 4B and 4C), indicating that lncRNA16 played a role in promoting progression of the cell cycle, probably at the G2/M checkpoint.

### LncRNA16 regulates the expression of cyclin B1

**Figure 5:**
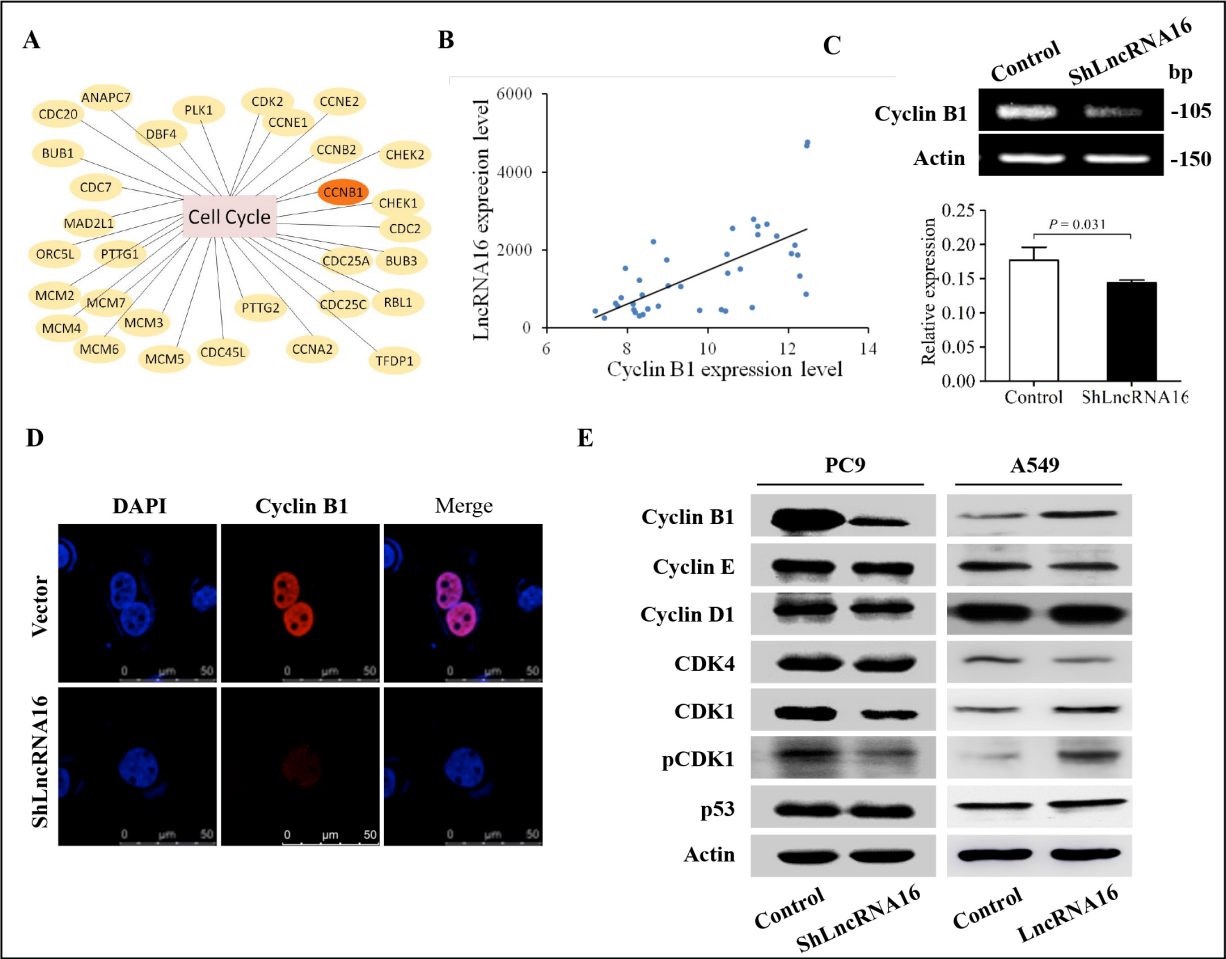
LncRNA16 increases expression of cyclin B1, CDK1 and pCDK1 expression was inhibited at the protein level (**A**) mRNA expression profiling revealed differential expression of cell cycle-related mRNAs. (**B**) Correlation between levels of cyclin B1 mRNA and lncRNA16 in 20 lung cancer tissues and adjacent matched normal tissues, as determined using Spearman correlation analysis. (**C**) Levels of cyclin B1 mRNA in PC9-shLncRNA16 and control cells were tested using RT-PCR (upper panel) and qRT-PCR (lower panel). (**D**) Cyclin B1 protein levels and localization in PC9-shLncRNA16 and control cells were analyzed by immunofluorescence assay. (**E**) Cyclin B1, CDK1 and pCDK1 are determined by Western blotting (WB). E Left, PC9 cells; E right, A549 cell.

As knockdown of lncRNA16 induced G2/M phase arrest, the molecular mechanisms underlying this process were investigated. For this, cell cycle-related mRNAs differentially expressed in our mRNA expression profile (Figure 5A). The results showed that of all the mRNAs, expression of cyclin B1 was positively correlated with levels of lncRNA16 in 20 lung cancer tissues and adjacent matched normal tissues (Figure 5B). In addition, mRNA levels of cyclin B1 decreased following lncRNA16 knockdown in PC9 cells (Figure 5C). Analysis by immunofluorescence assay showed a moderate decrease in nuclear-located cyclin B1 in PC9-shLncRNA16 cells (Figure 5D), indicating that upon reduction of lncRNA16, the amount of cyclin B1 that could translocate into the nucleus was also reduced. Therefore, the level of cyclin B1 protein and several other proteins involved in the cell cycle were measured in the PC9 cell line (Figure 5E left). Levels of cyclin B1, cyclin-dependent kinase 1 (CDK1), and pCDK1 proteins decreased with lncRNA16 knockdown in PC9 cells, whereas levels of other cell cycle-related proteins remained unchanged. It was reported that p53 regulated the expression of cyclin B1, however, our results showed that the expression of p53 was not changed by knocked-down lncRNA16. This result indicated that lncRNA16 regulated cyclin B1 independent with p53.

Consistent with knockdown of lncRNA16, we evaluated the effect of overexpression of lncRNA16 in A549 cells (Figure 5E right). Levels of cyclin B1, CDK1 and pCDK1 proteins increased with lncRNA16 overexpression in A549, whereas levels of other cell cycle-related proteins and p53 remained unchanged. These data indicated that lncRNA16 regulated G2/M associated proteins.

## DISCUSSION

By examining the differential expression of lncRNAs in lung cancer tissues and adjacent matched normal tissues, we identified lncRNA16 as a potential biomarker for early diagnosis of lung cancer. Specifically, we found that lncRNA16 was highly expressed in the lung cancer tissues, and its levels were significantly elevated in plasma of patients with lung cancer patients, even in the early stage.

Determination of blood-fluid tumor markers is a simple, inexpensive, and non-invasive test that greatly facilitates early diagnosis of cancer. However, in the case of lung cancer, none of the current biomarkers used in clinical settings are sensitive or specific enough for reliable cancer screening [12]. Therefore, identification of novel biomarkers for the early diagnosis of lung cancer will have significant clinical benefits. In a previous study, MALAT1 was reported as a potential complementary blood-based biomarker for the diagnosis of non-small cell lung cancer [28]. In another study, colon cancer-associated transcript 2 (CCAT2) was reported to promote invasion of non-small cell lung cancer, and had the potential as a biomarker for lymph node metastasis [29]. Our study indicated that lncRNA16 could be included in this short list of potential cancer biomarkers for early stage diagnosis of lung cancer. Furthermore, our AUC-ROC analysis showed that lncRNA16 was a suitable plasma biomarker for lung cancer diagnosis due to its high sensitivity and specificity.

The fact that cells uncontrollably enter the cell cycle is a hallmark feature of all tumors; hence, the genes that regulate the cell cycle, such as cyclins and CDKs, play an important role in cancer initiation and development [30–32]. One of the cyclins, cyclin B1, controls the transition from the G2 to M phase of the cell cycle, and abnormal expression of this protein is widely reported in cancers [33–35]. Our results indicated that lncRNA16 affected the G2/M phase by regulating the expression of cyclin B1 at the mRNA level in PC9 cells. Earlier studies have shown that p53 controls the G2 checkpoint by regulating the expression of cyclin B1 [36, 37]. However, in lung cancer, p53 did not regulate cyclin B1 because of its high mutational frequency. Conversely, this study showed that lncRNAs regulated expression of cyclin B1, probably in a manner independent of p53. These results suggested that lncRNA16 might be a promising biomarker for early diagnosis of lung cancer.

In conclusion, lncRNA16 was identified as a potential biomarker for lung cancer diagnosis, as it displayed significantly elevated levels over the baseline in patients with lung patient. Furthermore, we showed that the rate of false-negative results was significantly lower than that of markers used widely for lung cancer assessment. Further investigation will be required to elucidate the precise mechanisms of its action on cell cycle-related proteins, both at the level of inhibiting transcription and translation.

## MATERIALS AND METHODS

### Clinical samples and total RNA isolation

Patient data were collected from Peking University Cancer Hospital. We obtained 118 samples of cancerous tissues and adjacent matched normal tissues (at a distance of 5 cm from the tumor) from patients with lung cancer. In addition, 84 plasma samples of patients with lung cancer and 21 plasma samples from non-cancer participants (control samples) were collected. Informed consent was obtained from each subject or the subject's guardian. Acquisition and use of samples collected from the cohort was approved by the Ethics Committee of the hospital. Samples were stored at −80°C until further use. Total RNA was isolated from tissues and cells using TRIzol^®^ Reagent (Invitrogen, USA), while RNA in plasma was extracted using the Total Exosome RNA & Protein Isolation Kit (Invitrogen, USA), according to the manufacturer's instructions. After extraction, genomic DNA was removed from the isolated RNA using RQ1 RNase-Free DNase (Promega, USA), according to the manufacturer's instructions.

### The custom designed microarray platform

The custom designed microarray platform was manufactured by Agilent, consisting of probes for 39,311 lncRNA transcripts. At least one probe was designed for each lncRNA transcript. Of all of the probes designed for lncRNA transcripts, 28,937 are specific for lncRNA transcripts and do not overlap with protein coding loci.

### Quantification of lncRNA fragments by qRT-PCR

For lncRNA detection, the extracted RNA was reverse-transcribed into cDNA with random hexamer primers using the SuperScript^®^ III First-Strand Synthesis System for RT-PCR (Invitrogen, USA). qRT-PCR was performed using these cDNA products with SYBR^®^ Green PCR Master Mix (TransGen, China) on an Applied Biosystems 7500 Real-Time PCR system (Applied Biosystems, USA), and β-actin was used as an internal control. The sequences of the gene-specific primers were as follows. β-actin sense: 5′-TTAGTTGCGTTACACCCTT T-3′; antisense: 5′-ACCTTCACCGTTCCAGTTT-3′; LncRNA16: sense: 5′-GATGACAGTCTGCCTCTATCT TAC-3′; antisense: 5′-CTTTGAGCCAAGCAGGTTAT TG-3′. Relative lncRNA levels were calculated by 2^–ΔCT^ (where ΔCt = Ct(gene) − Ct(β-actin)). The fold change of lncRNA expression in tumor samples versus non-tumor samples was calculated using the 2^–ΔΔCT^ method.

### Cell culture and transfection

The lung cancer cell lines, PC9 and A549, were cultured at 37°C in Dulbecco's modified Eagle's medium containing 10% fetal bovine serum, in a humidified incubator containing 5% CO_2_. The PC9 cell line was authenticated by DNA sequencing with 3100 DNA Analyzer (Applied Biosystems, USA). LncRNA16-shRNA (GenePharma, Shanghai, China) and empty vector, lncRNA16 and empty vector were transfected. The sequence for lncRNA16-shRNA were synthesized commercially, and were of sequences: 5′-GCCAGCGUU ACAGUAAUGUTT-3′ (sense primer); and 5′-ACAUUAC UGUAACGCUGGCTT-3′ (antisense primer). After 6 h, the transfection media was replaced with normal media, and transfected cells were cultured for another 24 h. The transfected cells were selected in medium containing puromycin.

### Cell proliferation assay

An MTT assay was used (according to the manufacturer's instructions) to detect cell proliferation. First, 2 × 10^3^ cells were seeded on 96-well culture plates. After 24 h, MTT was added to each well and the plate was incubated at 37°C for an additional 4 h. Subsequently, the media was replaced with dimethylsulfoxide (DMSO), and samples were shaken for 15 min to dissolve the formazan crystals formed. A microplate spectrophotometer was used to measure absorbance at 490 nm. Each MTT assay was repeated at least three times. To determine colony formation, 500 cells were placed in each 60-mm plate and cultured in media containing 10%FBS, with the medium being replaced every 4 days. After two weeks of culturing, cells were fixed with 75% methanol and stained with 0.1% crystal violet. Visible colonies were counted manually. Each experiment was performed three times.

### Flow cytometric analysis

Cell cycle analysis was performed using a FACScan flow cytometer. When the cells had grown to 70–80% confluence, they were collected, washed twice with PBS, and then fixed overnight in 70% ethanol at 4°C. After fixation, the cells were washed with PBS and resuspended in 300 mL of PBS containing 0.25 mg/mL RNase A, followed by incubation at 37°C for 30 min. Subsequently, propidium iodide was added at a final concentration of 0.05 mg/mL, and samples were incubated for 15 min at room temperature in the dark, before flow cytometer analysis. For each sample, 2 × 10^4^ events were acquired for further analysis.

### Western blot

Cells were initially lysed in 1 × SDS loading buffer containing protease inhibitors. Next, 30 μg of cell lysates were subjected to SDSPAGE, and then transferred to polyvinylidenedifluoride (PVDF) membranes. These were incubated with specific antibodies for β-actin (1:10^4^, Sigma), cyclin B1 (1:2000, Affinity), cyclin E (1:2000, NeoMarker), cyclin D1 (1:2000, Santa Cruz), cyclin-dependent kinase 1 (CDK1) (1:2000, Santa Cruz), p-CDK1 (1:1000, Santa Cruz), cyclin-dependent kinase 4 (CDK4) (1:2000, Santa Cruz) and p53 (1:3000, Santa Cruz), and visualized by using a SmartChemi^TM^ Image Analysis System (Beijing Sage Creation Science, China).

### Immunofluorescence

Cells were placed on glass coverslips in 35-mm plates and fixed with 4% paraformaldehyde for 30 min before being incubation in 0.5% Triton X-100 for 15 min. After blocking with 5% bovine serum albumin for 1 h, cells were incubated with rabbit polyclonal anti-cyclin B1 antibody (1:100, Affinity) and TRITC-conjugated goat anti-rabbit IgG antibody. DAPI was used for observation of cell nuclei.

### Tumor growth in nude mice

Four-week-old female immunodeficient nude BALB/c mice were purchased from Vital River Laboratories (VRL) and bred at the animal center of Peking University Cancer Hospital & Institute. Cells were harvested, washed with PBS, and re-suspended in 1 × PBS. Subsequently, 5 × 10^6^ cells (0.1 mL) were injected subcutaneously into the abdomen of mice. Tumor size was measured every week. Tumor volume was calculated according to the following formula: volume = width^2^ × length/2. Animal experiments were approved by the Animal Ethics Committee of the hospital research department.

### Statistical analysis

All data were analyzed with the statistical package SPSS17.0, and data are presented as mean ± SD. The Student's *t* test, Chi square test, and Mann–Whitney test were used to determine the significant of differences between two groups. *P* values < 0.05 were considered statistically significant.
